# Gene Expression Mapping of Histone Deacetylases and Co-factors, and Correlation with Survival Time and ^1^H-HRMAS Metabolomic Profile in Human Gliomas

**DOI:** 10.1038/srep09087

**Published:** 2015-03-20

**Authors:** Nassim Dali-Youcef, Sébastien Froelich, François-Marie Moussallieh, Salvatore Chibbaro, Georges Noël, Izzie J. Namer, Sami Heikkinen, Johan Auwerx

**Affiliations:** 1 Laboratoire de Biochimie et Biologie Moléculaire, Hôpitaux Universitaires de Strasbourg, Nouvel Hôpital Civil, 1 place de l'hôpital, 67091 Strasbourg Cedex, France; 2 Institut de Génétique et de Biologie Moléculaire et Cellulaire (IGBMC)/CNRS UMR 7104/INSERM U 964/Université de Strasbourg, 1 rue Laurent Fries, 67404 Illkirch, France; 3 Institut Clinique de la Souris, 1 rue Laurent Fries, 67404 Illkirch, France; 4 Department of Neurosurgery, Hôpitaux Universitaires de Strasbourg, avenue Molière, 67085 Strasbourg Cedex, France; 5 Centre Paul Strauss, 3 rue de la porte de l'Hôpital 67065 Strasbourg, Cedex, France; 6 Department of Biophysics and Nuclear Medicine, Hôpitaux Universitaires de Strasbourg, CHU de Hautepierre, avenue Molière, 67200 Strasbourg Cedex, France

## Abstract

Primary brain tumors are presently classified based on imaging and histopathological techniques, which remains unsatisfaying. We profiled here by quantitative real time PCR (qRT-PCR) the transcripts of eighteen histone deacetylases (HDACs) and a subset of transcriptional co-factors in non-tumoral brain samples from 15 patients operated for epilepsia and in brain tumor samples from 50 patients diagnosed with grade II oligodendrogliomas (ODII, n = 9), grade III oligodendrogliomas (ODIII, n = 22) and glioblastomas (GL, n = 19). Co-factor transcripts were significantly different in tumors as compared to non-tumoral samples and distinguished different molecular subgroups of brain tumors, regardless of tumor grade. Among all patients studied, the expression of *HDAC1* and *HDAC3* was inversely correlated with survival, whereas the expression of *HDAC4*, *HDAC5*, *HDAC6*, *HDAC11* and *SIRT1* was significantly and positively correlated with survival time of patients with gliomas. ^1^H-HRMAS technology revealed metabolomically distinct groups according to the expression of HDAC1, HDAC4 and SIRT1, suggesting that these genes may play an important role in regulating brain tumorigenesis and cancer progression. Our study hence identified different molecular fingerprints for subgroups of histopathologically similar brain tumors that may enable the prediction of outcome based on the expression level of co-factor genes and could allow customization of treatment.

Primary brain tumors are notoriously heterogenous. The overall mortality rate for some tumor subtypes, such as high-grade gliomas, remains high and only limited progress in survival has been achieved in the last decades^1^. Furthermore, the responsiveness of patients to therapeutic protocols varies with no clear predictive factor to determine treatment response. An in-depth dissection of the molecular basis of resistance to treatment and the identification of specific signaling pathways that are involved in tumor development and progression, are key in the search for a more targeted and effective therapies for brain tumors.

Perhaps equally important to the numerous genetic mutations, amplifications and deletions that accumulate in a given tumor are epigenetic modifications^2^. One of the best-characterized epigenetic modifications is the acetylation/deacetylation status of lysine residues in histones, which regulates gene transcription by controlling chromatin unfolding (activation) and condensation (silencing), respectively. These two opposing processes are regulated through histone acetyltransferases (HATs) and histone deacetylases (HDACs) (for review see Refs. 3,4,5), respectively. The HDAC family of lysine deacetylases are homologs of the yeast Rpd3/Hda1 deacetylases and contain 11 members termed *HDAC1-11* that divide into 3 classes I, II and IV. Class III consists of the sirtuin family of HDACs represented by 7 members (SIRT1-7) sharing a 270 aminoacid catalytic domain and a mandatory metabolic co-factor, NAD^+^ (reviewed in Ref. 6).

Several HATs and HDACs have been implicated in tumorigenesis, and HDAC inhibitors (HDACi) effectively inhibit tumor growth and progression^7^,^8^ (a review). Class I and II HDACs are generally located in the nucleus and are overexpressed in various cancers^9^,^10^ (reviews)^11^,^12^,^13^,^14^,^15^. Furthermore, class II and IV HDACs were recently shown to be downregulated in glioblastomas^16^. A set of sirtuin genes, including *SIRT1*, *SIRT3* and *SIRT7*, are overexpressed in certain cancers^17^ (a review). The role of SIRT1 in cancer is not fully understood and remains controversial. For instance, when SIRT1 was shown to suppress tumorigenesis in colon cancer growth through deacetylation of β-catenin^18^, *SIRT1*-knockdown significantly delayed mitotic entry of glioma cells, inhibited cell growth and proliferation, and promoted apoptosis^19^. In addition, *SIRT1* knockdown sensitized glioma cells to radiation-induced apoptosis^20^. Recently, SIRT1 was suggested to mediate cell proliferation in gliomas through the connection with the forkhead box M1^21^. In line with the latest cited studies, we have recently shown that specific intestinal loss of SIRT1 protects from colitis-associated colorectal cancer^22^. Finally, the expression of the « checkpoint » tubulin deacetylase SIRT2 was reported to be reduced in gliomas^23^,^24^ and was shown to be required for glioma stem cell proliferation arrest^25^.

Because of the genetic heterogeneity and the poor treatment responsiveness, a diagnostic approach that establishes the molecular signature for the tumor would be highly beneficial. To this end, we measured the expression of the 18 known HDAC genes and of 6 genes encoding transciptional co-factors in 50 samples representing 3 histopathologically identified gliomas, namely W.H.O. (World Health Organization) grade II oliodendrogliomas (ODII), grade III oliodendrogliomas (ODIII), grade IV glioblastoma (GL), and in 15 control brain samples from epilepsia-operated patients. Cluster analysis of the gene expression data enabled the identification of molecular subgroups of tumors that were undistinguishable by histopathological methods.

## Results

### Expression pattern of HDACs in brain tumors by qRT-PCR

**Figure 1:**
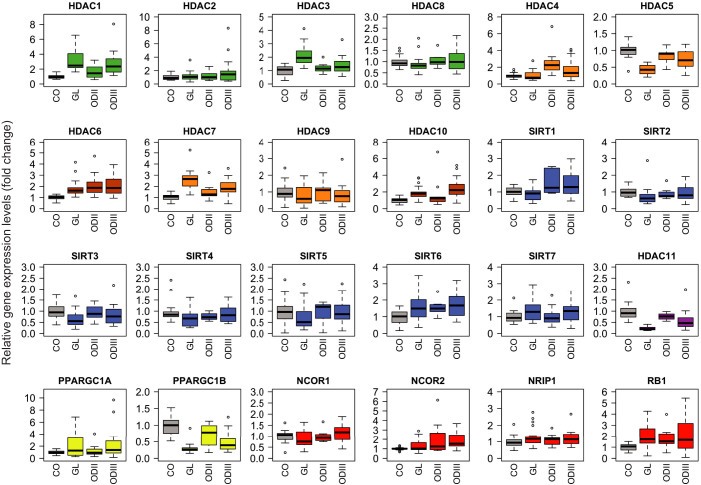
Quantitative expression of the 18 histone deacetylases (HDACs) and 6 metabolic cofactors in different human brain tumors as indicated. The qRT-PCR data are presented as medians of the gene expression level normalized first to the 18S gene then to non-tumoral specimen (the median of controls was set to 1). The boxplots represent the expression rates ranging from the lowest to the highest value. Outlier values are also represented.

**Figure 2:**
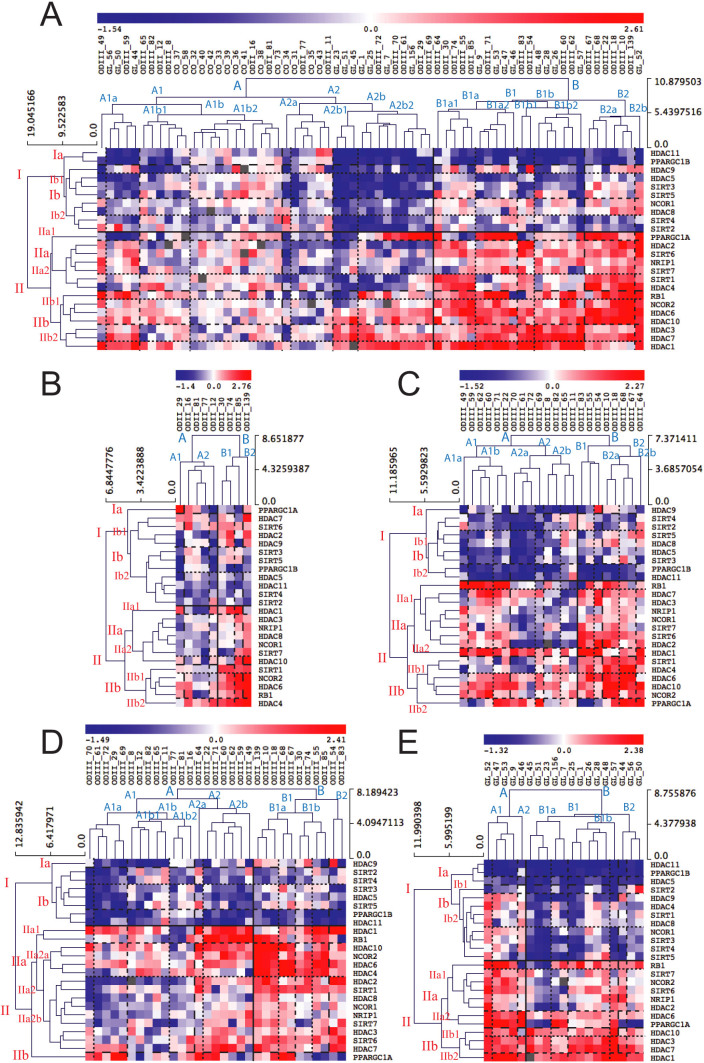
(A) Unsupervised hierarchical clustering of HDAC and metabolic cofactor gene expression profiles in human brain tumor and non-tumoral (controls) samples. Subgroups identified by cluster analysis are highlighted by horizontal lines for genes and by vertical lines for brain tumor samples. Increased gene expression is represented by the red color and decreased expression in blue. The intensity of the color represents the degree of expression. The same clustering analysis was performed separately for ODII tumor samples (B), ODIII (C), ODII and ODIII together (D) and GL (E).

To characterize the molecular fingerprint of brain tumors, we analyzed by qRT-PCR the expression profile of all HDACs (class I–IV) and six transcriptional co-factors in different brain tumor samples and control brain tissues. All HDACs were expressed in non-tumoral samples, and their expression was altered in many tumor types (Figure 1). For clarity, the levels of significance are presented separately in Table S2. The degree of increase in *HDAC1* expression followed the grade of gliomas. *HDAC1* levels were significantly increased in ODIII and grade IV gliomas, while the increase in ODII did not reach statistical significance. *HDAC2* was increased only in ODIII (1.9-fold, p = 0.051). *HDAC3* was significantly increased only in malignant ODIII and GL gliomas. *HDAC4* was significantly increased in ODII and ODIII but the expression in glioblastoma was heterogenous among this class of patients and overall did not reach statistical significance (Figure 1 and Table S2). *HDAC6* expression was significantly increased in all grades of gliomas, whereas *HDAC7* and *HDAC10* were significantly increased only in ODIII and GL tumors. *HDAC8* and *HDAC9* expression levels were not significantly changed in gliomas as compared to control patients. Among class I and II HDACs, *HDAC5* was particular and its expression was significantly decreased in ODIII and GL tumors. Class IV *HDAC11* seemed to follow a tumor grade gradient in its decrease. Hence, *HDAC11* expression was significantly decreased in ODIII tumors and dramatically diminished in GL patients. The decrease in *HDAC11* expression in ODII patients did not reach statistical significance. Regarding the sirtuin family of class III HDACs, *SIRT1* expression was moderately but significantly increased in ODII and ODIII while it was modestly diminished in GL, yet this decrease was not statistically significant. *SIRT2* expression was significantly decreased in most of GL patients with the exception of one patient who had an increased expression. *SIRT2* expression was heterogeneous in ODII and ODIII gliomas, a subset of patients exhibiting a moderate increased expression, while most of them had decreased expression levels. *SIRT3* expression was significantly decreased in the aggressive GL tumors but the decrease was not significant in grade II and III gliomas. *SIRT4* and *SIRT5* expression was decreased in most of the patients, but the decrease was not statistically significant due to an increased expression in few patients (Figure 1 and Figure 2). Conversely, *SIRT6* expression was moderately and significantly increased in all gliomas. As for *SIRT7*, the modest increased expression in ODIII and GL tumors was not statistically significant. Regarding the metabolic co-activators PGC1α and PGC1β (encoded by the *PPAGC1A* and *PPARGC1B* genes, respectively), *PPARGC1A* expression was significantly increased in ODIII and GL tumors, whereas *PPARGC1B* expression was robustly decreased in all gliomas. In average, the decrease in *PPARGC1B* expression level followed a tumor gradient, grade IV tumors exhibiting the lowest expression. Among the co-repressors, only *NCOR2/SMRT* was significantly increased in ODIII tumors. The expression of the tumor suppressor gene *RB1* was moderately increased in ODII tumors (p = 0.06) and significantly enhanced in grade III and IV gliomas (p < 0.01).

### Cluster analysis of HDAC and NR co-factor expression profiles

Cluster analysis is suited to identify groups of genes that are co-regulated and potentially share functional commonalities. Illustrating this principle, cluster analysis distinguished three main groups of genes based on their expression levels in all tumor types, regardless of the HDAC classification. Of particular note, non-tumoral samples (CO) clustered together and were separated from the tumor samples (Figure 2A). Gene cluster I represents genes that were mostly down-regulated in a subgroup of gliomas (A2b), including *HDAC11*, *PPARGC1B*, *HDAC9*, *HDAC5*, *SIRT3*, *SIRT5*, *NCOR1*, *HDAC8*, *SIRT4*, and *SIRT2*. In subgroup B, two genes *HDAC11* and *PPARGC1B* were down-regulated in all tumors, suggesting that these co-factors might regulate genes commonly induced in tumorigenesis (e.g. anti-apoptotic and proliferation genes, or genes involved in oxidative metabolism). Other cluster I genes showed heterogeneous expression among the gliomas belonging to this subgroup (Figure 2A). Gene cluster II represents genes that were mostly overexpressed in a subgroup of tumors (B) and include *RB1*, *NCOR2*, *HDAC6*, *HDAC10*, *HDAC3*, *HDAC7* and *HDAC1*. In this cluster, *HDAC1* was the only gene that was induced in almost all tumor samples, suggesting that *HDAC1* play an important role in brain tumorigenesis (Figure 2A). The metabolic regulator *PPARGC1A* (subcluster IIa) was variably expressed among tumors. Some tumors displayed a robust overexpression of *PPARGC1A* while other samples showed a strong down-regulation or only a modest change in expression. In addition, two genes *SIRT1* and *HDAC4* that clustered together (in gene cluster IIa) were strongly overexpressed in two subgroups of tumors (B1a1 and B2) but significantly down-regulated in most of the samples of subgroup A2b.

Panels B to E in Figure 2 represent gene clusters in specific tumor types (ODII, ODIII, ODs and GL). One can appreciate that genes cluster differently in different tumor types that represent different tumor grades, suggesting different molecular signatures for each type or grade. Secondarily, some genes are overexpressed in a group of patients while down-regulated or unchanged in others within the same histopathologic entity, underscoring the molecular heterogeneity of tumors that are indistinguishable by histopathologic means. Finally, within OD tumors, we observed that ODII tumors do not necessarily form a distinguished cluster from ODIII tumors (Figure 2D).

In summary, HDAC and co-factors gene expression levels discriminate subgroups of patients that do not necessarily belong to the same histopathologic grade (*e.g.* some ODIII cluster with GL tumors, Figure 2A) and even within the same grade, molecular subgroups were identifiable, which challenges the histopathological classification and might explain why all glioma patients do not respond equally to the available therapeutic protocols.

### Pearson correlation between the expression level of HDACs and cofactors among gliomas

**Figure 3:**
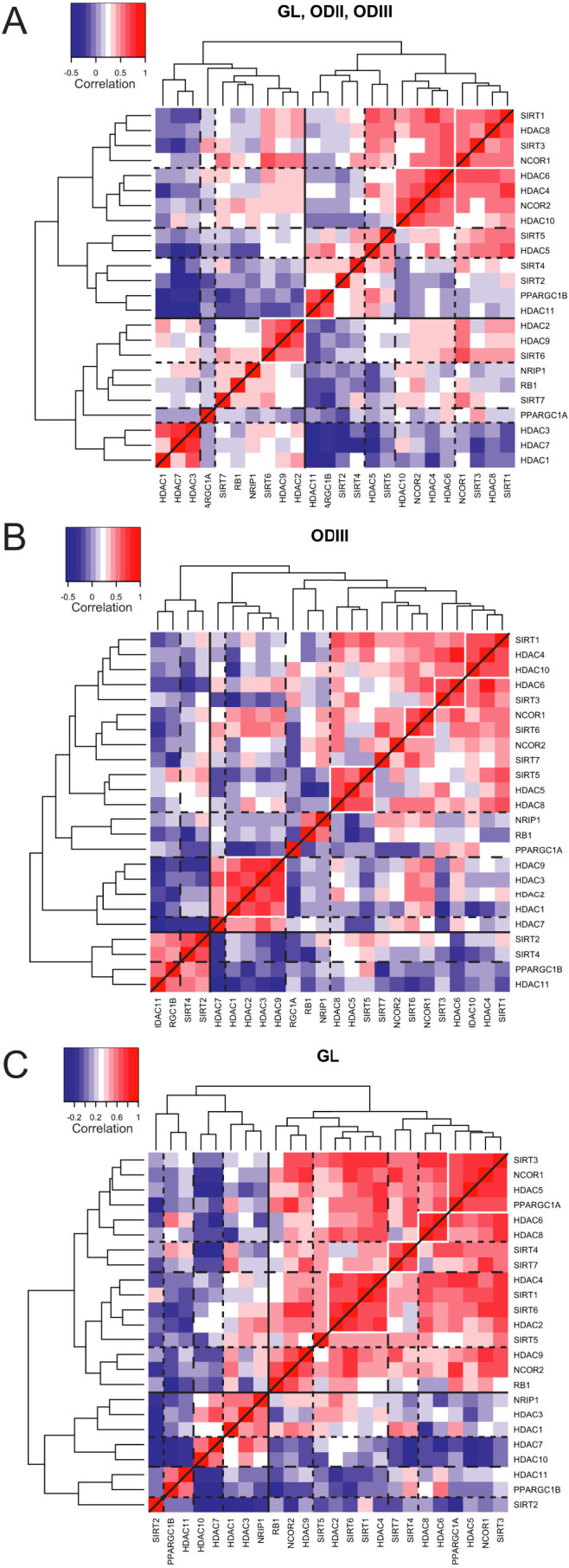
Pairwise-Pearson correlation (PPC) analysis of HDACs and co-factors in glioma samples. Unsupervised hierarchical clustering was carried out on pairwise Pearson gene-to-gene correlations using heatmap.2 from the R package gplots. Correlation coefficient equal to 1 represents the correlation of each gene to itself and is shown in red with the highest intensity. Negative correlations are shown in a light (poor correlation) to dark (strong correlation) blue color scale. The analysis was performed for all tumors together (GL, ODII and ODIII) (A), or separately for ODIII (B) and GL (C).

We derived a heatmap from the gene-to-gene Pearson correlations for the 24 HDACs/cofactors within our qRT-PCR expression profiles in all gliomas, i.e. GL, ODII and ODIII together (Figure 3A). Among the positively co-correlated genes, *HDAC1*, *HDAC3* and *HDAC7* formed the clearest cluster. Other clusters with positively co-correlated genes contained *PPARGC1B* and *HDAC11*, both strongly down-regulated in gliomas (Figure 1), and *HDAC2*, *HDAC9* and *SIRT6*. Yet another major positively co-correlated cluster was identified, containing two subclusters of *SIRT1*, *HDAC8*, *SIRT3* and *NCOR1* genes on one hand and *HDAC6*, *HDAC4*, *NCOR2* and *HDAC10* genes on the other. The strongest correlation within this cluster was that between *SIRT1* and *HDAC4* genes. We also identified clusters of genes that were negatively co-correlated among glioma patients. Hence, we identified *HDAC3*, *HDAC7* and *HDAC1* that were found to be robustly and negatively correlated with *HDAC11*, *PPARGC1B*, *SIRT2* and *SIRT4*, with the exception of *SIRT4* and *HDAC1* that were not correlated. This was also the case, though to a lesser extent, for the negative correlations found between the members of the *SIRT1*, *HDAC8* and *SIRT3* cluster and the members of the *HDAC1*, *HDAC7* and *HDAC3* cluster (Figure 3A). We then analyzed the gene-to-gene correlations separately in the most aggressive gliomas. Amongst the ODIII samples, the strongest positive correlations were within cluster 1: *SIRT1*, *HDAC4* and *HDAC10*; cluster 2: *HDAC6* and *SIRT3*; cluster 3: *NCOR1* and *SIRT6*; cluster 4: *SIRT5*, *HDAC5* and *HDAC8*; and cluster 5: *HDAC9*, *HDAC3*, *HDAC2* and *HDAC1* (Figure 3B). For GL, the strongest positive correlations were within cluster 1: *SIRT3*, *NCOR1*, *HDAC5* and *PPARGC1A*; cluster 2: *HDAC6* and *HDAC8*; cluster 3: *SIRT4* and *SIRT7*; and cluster 4: *HDAC4*, *SIRT1*, *SIRT6* and *HDAC2* (Figure 3C). Of particular note, also *RB1*, *NCOR2* and *HDAC9* were positively correlated indicating that these genes might interact and participate in the pathogenesis of glioblastoma.

These gene-to-gene correlations among HDACs and cofactors might reveal transcriptional cues for other genes involved in a particular signaling pathway involved in the pathogenesis of these tumors. Other experiments are needed to verify the potential physical interaction between the co-correlated genes in the context of brain tumorigenesis.

### Correlation of gene expression profile of HDACs and cofactors with survival of patients with gliomas

**Figure 4:**
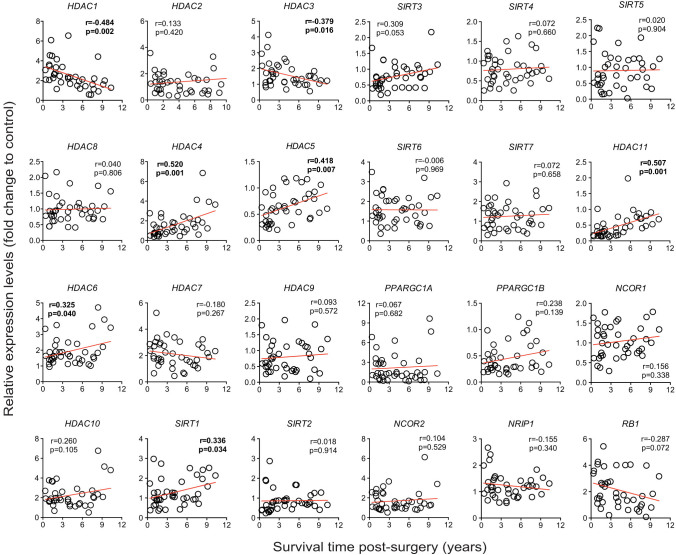
Correlation of relative gene expression levels in glioma tumor samples to the time of survival of patients. Correlations that reached statistical significance are in bold.

In order to evaluate the potential for the analyzed 24 HDAC and cofactor genes to serve as markers of how aggressive the gliomas are, we correlated the tumor expression profiles of these genes with the survival time of those 40 patients from whom the tumor samples were obtained (and whom had died during the study period). Of all the genes studied, *HDAC1* and *HDAC3* were inversely and significantly correlated with the survival of patients with gliomas, when analyzed collectively (p = 0.002 and p = 0.016 respectively, Figure 4). *RB1* expression levels tended to correlate negatively with survival of patients, although the correlation did not reach statistical significance (p = 0.072, Figure 4). *HDAC4*, *HDAC5*, *HDAC6* and *HDAC11* expression levels were positively correlated with survival (p = 0.001, p = 0.007, p = 0.04 and p = 0.001 respectively, Figure 4). Among the sirtuin family of HDACs, only *SIRT1* correlated significantly with the survival of patients (p = 0.034, Figure 4), while a tendency for SIRT3 was observed (p = 0.053, Figure 4).

Interestingly, when samples were analyzed separately based on tumor grade, *HDAC11* expression levels correlated significantly with survival time among GL patients (p = 0.004, n = 15) but not among ODIII patients (p = 0.198, n = 20), suggesting that the decreased expression of *HDAC11* in grade IV tumors (i.e. GL) may have a greater impact on tumor aggressiveness and clinical outcome than in the less aggressive grade III oligodendroglioma. Of note, when analyzing ODIII and GL together, *HDAC11* still correlated with survival time of patients (p = 0.009, n = 35). As for *HDAC4* and *HDAC6*, the opposite was observed. These genes were correlated with survival time in ODIII patients (p = 0.006 for HDAC4 and p = 0.047 for HDAC6, n = 20) but they did not in GL patients (p = 0.552 for HDAC4 and p = 0.381 for HDAC6, n = 15), suggesting that *HDAC4* and *HDAC6* may play a greater role in ODIII tumor aggressiveness than in GL tumors.

**Figure 5:**
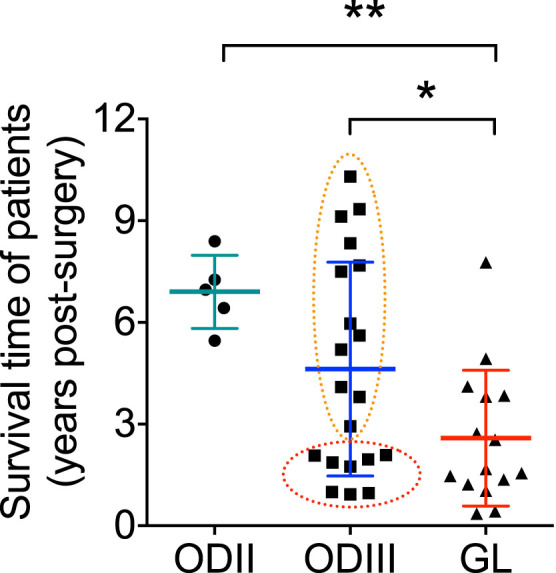
Mean survival time in patients with grade II, III and IV gliomas. The survival time was determined from the time of surgery until the death of the patient. The orange dashed ellipse represents a distinguished set of histopathological classified ODIII patients whose survival time were much higher from the rest of ODIII patients with poor survival time (red ellipse; some of these tumors were even more aggressive than some grade IV GL tumors). * p < 0.05, ** p < 0.01.

We then analyzed the mean survival times of patients between the tumor grades. As expected, we observed a clear inverse relationship between the grade of the tumor and the survival time (Figure 5). Interestingly, based on the survival time of patients, the ODIII group was heterogeneous and divided in two groups. One subgroup had a high survival time with an average survival time similar to that of the ODII group (surrounded by the orange dashed ellipse in Figure 5), the second group had a low survival time (red dashed ellipse in Figure 5) similar to the GL patients and even more aggressive in average to the mean survival time of GL.

From this survival analysis, we can conclude that of all the HDACs and co-factors analyzed, the expression levels of only a subset of genes correlated significantly with the post-surgery survival time of glioma patients who died during the study period. Furthermore, tumor aggressiveness and survival may be significantly affected by the expression of just one gene (*HDAC11* in GL and *HDAC4* and *HDAC6* in ODIII). Finally, the molecular heterogeneity observed in cluster analysis amongst OD tumors, together with the fact that the mean survival time of a subgroup of ODIII was similar to ODII patients, suggests that the histopathological classification of patients with gliomas does not necessarily follow the level of aggressiveness of the tumors.

### ^1^H-HRMAS analysis of brain tumors

**Figure 6:**
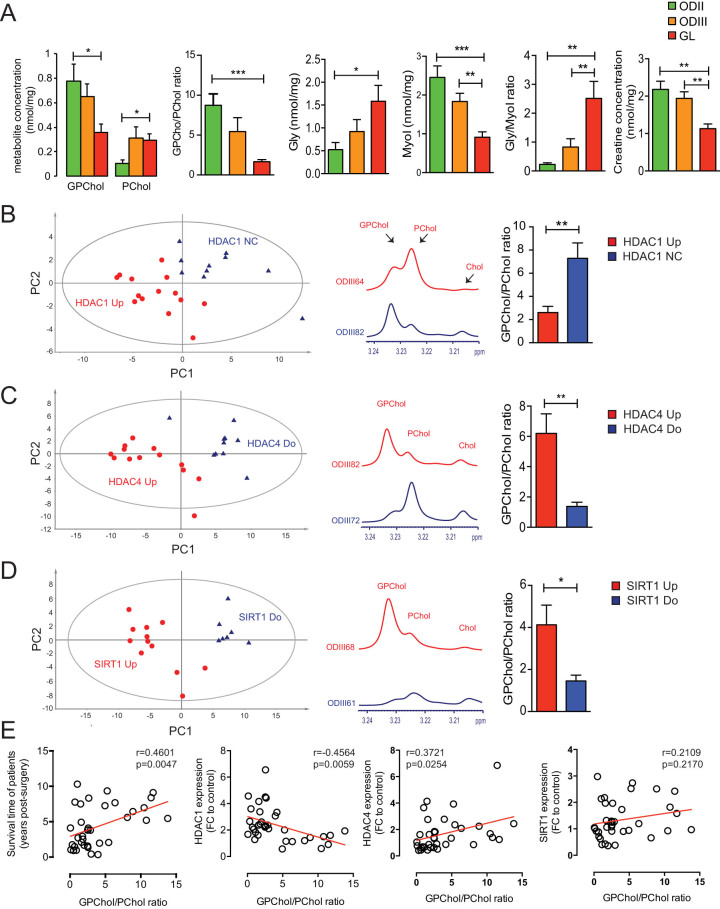
^1^H-HRMAS NMR spectroscopy analysis of grade II–IV gliomas. (A) HRMAS-measured glycerophosphocholine (GPChol), phosphocholine (PChol), glycine (Gly), myo-inositol (MyoI) and creatine (Cr) metabolites in grade II (ODII, n = 9), grade III (ODIII, n = 19) and grade IV (glioblastoma, GL, n = 17). The GPChol/PChol and Gly/MyoI ratios are also presented. (B) Results of two-component PLS-DA model built on the following metabolites: Glycine (Gly), glutamate, aspartate, serine, N-acetyl aspartate, acetate, succinate, glycerophosphocholine (GPChol), phosphocholine (PChol), lactate, isoleucine, valine, reduced glutathione (GSH), creatine (Cr), ascorbate, lysine, myo-inositol (MyoI), alanine, taurine and glutamine among oligodendrogliomas (ODII and ODIII) according HDAC1 expression level. HDAC1-overexpressing tumors (HDAC1 Up, red dots) and samples with unchanged HDAC1 expression (HDAC1 NC, blue triangles) formed metabolomically distinct classes. (C). Results of 2-class PLS-DA model among gliomas (regardless of tumor grade) according to HDAC4 expression level. HDAC4-overexpressing samples (HDAC4 Up, red dots) and samples with downregulated HDAC4 (HDAC4 Do, blue triangles) form two different groups. (D) 2-class PLS-DA model according to SIRT1 expression level in glioma samples. SIRT1-overexpressing samples (SIRT1 Up, red dots) and samples with downregulated SIRT1 (SIRT1 Do, blue triangles) form distinct groups. A zoom of representative spectra indicating the intensity of GPChol, PChol and choline (Cho) as well as the quantification of the GPChol/PChol ratio in each model are presented (B, C and D). E) Correlation of survival time, HDAC1, HDAC4 and SIRT1 expression with GPChol/PChol ratio among gliomas.

We used a metabolomic approach to compare the metabolomic profile of tumors based on their histological grade then on the assignation to different molecular subtypes for genes that were correlated to survival time among glioma patients. We will present only meaningful results. The statistical significance of the different group comparisons was assessed by the partial least square discriminant analysis (PLS-DA) statistical model, a supervised analysis procedure that maximizes the separation between classes based on their metabolomic profile. PLS-DA models were built for each hypothesis using the most discriminating binned variables (e.g. with a variable of importance value superior to the cut off of 1.0) corresponding to the most discriminating metabolites among the following list: Glycine (Gly), glutamate, aspartate, serine, N-acetyl aspartate, acetate, succinate, glycerophosphocholine (GPChol), phosphocholine (PChol), lactate, isoleucine, valine, reduced glutathione (GSH), creatine (Cr), ascorbate, lysine, myo-inositol (MyoI), alanine, taurine and glutamine. We presented in Figure 6A a quantification of the following metabolites, which concentrations have been shown to vary significantly in high grade brain tumors as compared to low grade tumors: PChol, GPChol, Gly, MyoI and Cr^26^,^27^,^28^. We observed that as the grade of tumor increased the GPChol/PChol ratio, MyoI and Cr decreased significantly, whereas Gly and Gly/MyoI ratio were significantly increased (Figure 6A). Since a decreased GPChol/PChol ratio seems to be associated with an increased malignancy and that the expression of some HDACs was correlated with survival, we stratified patients based on the expression of *HDAC1*, *HDAC4* and *SIRT1* regardless of tumor grade and compared their metabolomic profiles. Given that all GL patients had an increased *HDAC1* expression level, we ran the hypothesis only for ODs. We observed that OD samples with high *HDAC1* expression formed a metabolomically distinct group from patients with unchanged (NC = not changed) HDAC1 expression, the former having a significantly lower GPChol/PChol ratio (Figure 6B). Along the same line, glioma samples (ODs and GL) with high *HDAC4* and *SIRT1* expression were metabolomically different from samples with low *HDAC4* and *SIRT1* expression, the latter having a significantly lower GPChol/PChol ratio than the former group (Figure 6C and 6D, respectively). Of interest, we observed that the GPChol/PChol ratio correlated with survival time among our glioma patients (Figure 6E). Also, this ratio was negatively and positively correlated with *HDAC1* and *HDAC4* expression, respectively while the correlation with *SIRT1* expression did not reach statistical significance (Figure 6E).

Altogether, metabolomic data suggest that a decreased GPChol/PChol ratio is associated with high-grade tumors and that the expression of *HDAC1*, *HDAC4* and *SIRT1* may influence tumor aggressiveness through changes in metabolomic profiles of tumors.

## Discussion

The expression analysis of the 18 known HDACs and a subset of 6 transcriptional regulators in 50 brain tumors samples obtained from patients operated from grade II–IV gliomas, and 15 non-tumoral samples allowed us to discriminate brain tumors with indistinguishable histopathological features by their different molecular « fingerprints ». The relevance of studying the expression of HDACs in brain tumors lies in the fact that their overexpression has been documented in several cancers, and some HDAC inhibitors have shown clinical efficacy for the treatment of some cancers^29^,^30^,^31^. Of particular note, *HDAC5* was the only class II member whose expression was reduced in most tumors studied here (Figure 2A) and robustly so in all of our glioblastoma samples (Figure 2E). Interestingly, *HDAC5* overexpression in tumor cell lines has been shown to blunt tumor growth and promote apoptosis through the extrinsic pathway^32^, underpinning the tumor suppressor potential for *HDAC5*. In addition, we found a strong positive correlation between *HDAC5* expression levels and survival time of patients with gliomas (Figure 4), strengthening the notion that *HDAC5* may be an important factor in the epigenetic control of genes involved in tumor growth and apoptosis of cancer cells. Even though the expression level for *HDAC4* did not vary within the GL tumors, the expression of *HDAC4* correlates positively with a better survival time across all tumor samples (Figures 4). The biological significance of this observation needs further clarification.

Consistent with a recent study showing that class IV *HDAC11* was strongly down-regulated in GL tumors^16^, we found the expression of *HDAC11* diminished in all tumor samples, especially those with high grade of malignancy. In addition, we found that *HDAC11* expression levels correlated significantly with the survival time of patients with gliomas (all tumor samples combined). This was mainly the case for the most aggressive GL, as *HDAC11* expression level was not correlated with the survival time amongst ODIII patients, which could be due to the scattered distribution of survival time in the ODIII group (see Figure 5). In average, *HDAC11* expression decreased with the increase in the grade of the tumor (Figure 1). It has been reported that the disruption of *HDAC11* in antigen presenting cells (APCs) induces the expression of *IL10* and subsequently impairs antigen-specific T cell responses^33^. These data suggest that *HDAC11* may have an important role in modulating the immune response against gliomas, thereby promoting tumor growth suppression. Hence, HDAC11 inhibition below a certain threshold may not be sufficient to the immune system to fight against the tumor, which therefore becomes more aggressive affecting thereby the survival time of patients. Of particular note, it has been shown recently that *HDAC6* and *HDAC11*, which were expressed in opposite manner in our glioma samples (*HDAC6* overexpressed and *HDAC11* down-regulated), have divergent roles on the transcriptional regulation of *IL10* in antigen presenting cells^34^,^35^. In view of our data and given the role of *HDAC11* in antigen-specific T cell responses, we suggest that restoring *HDAC11* expression levels to normal values (e.g. through lentiviral-mediated gene therapy) may improve the survival of patients with gliomas, especially GL.

Regarding the SIRT genes, we observed that expression of the mitochondria-localized *SIRT3* was significantly downregulated in GL tumors in average, albeit not in all samples, suggesting a potential role of *SIRT3* as a discriminant gene in the molecular signature of brain tumors. *SIRT3* was previously described as a tumor suppressor gene, whose suppression induced an increase in glycolysis and a decrease in oxidative phosphorylation^36^. This is consistent with a potential anti-Warburg effect^37^ of *SIRT3*, knowing that aggressive tumors consume glucose at a higher rate than less aggressive ones to generate ATP. Interestingly, we found a modest positive correlation of *SIRT3* expression with survival of patients. This could be ascribed to the decreased expression of *SIRT3* in highly aggressive GL tumors (Figure 4).

Of particular interest, we found that *SIRT1* and *HDAC4* co-correlate in GL and in ODIII, suggesting that these genes cooperate to regulate a yet to be identified target gene or gene set. Two transcription factors, myocyte enhancer factor 2 (MEF2), involved in skeletal muscle differentiation, and hypermethylated in cancer 1 (HIC1), silenced in many human cancers, are regulated by SIRT1 and HDAC4 through deacetylation and sumoylation, respectively^38^,^39^. We showed that samples with overexpressed *HDAC4* and/or *SIRT1* were associated with an increased GPChol/PChol ratio. An increased choline kinase (which enhances PChol levels) and glycerophosphodiesterase activities (which decreases GPChol levels) have been linked to enhanced tumorigenesis and cancer cell migration^40^,^41^,^42^. These observations suggest that *SIRT1* and *HDAC4* may regulate the choline pathway to block tumorigenesis limiting thereby tumor aggressiveness and that their downregulation might predict a bad prognosis in gliomas patients.

It is known that reactive oxygen species (ROS)-mediated mtDNA mutations are associated with malignant cancer progression^43^. Overexpression of PGC-1 cofactors (α and β) was reported to protect cells against ROS by up-regulating anti-oxidant defense mechanisms^44^. It is tempting to speculate that the decrease in *PPARGC1B* observed in our brain tumor samples could participate in the increase in ROS formation and eventually impact on mtDNA mutations. However, the increase in the other *PPARGC1*, namely *PPARGC1A*, could be a compensatory mechanism to mitigate the *PPARGC1B* decrease and hence restrain ROS formation. Moreover, *PPARGC1A* and *PPARGC1B* have been shown to protect neurons from mitochondrial loss and oxidative damage^45^. Interestingly, *PPARGC1A* expression was differentially affected within our tumor sample of the same histopathological entity. The pathophysiological relevance of this differential expression in *PPARGC1A* needs to be determined. It is worth mentioning that we did not find any correlation between *PPARGC1A* and *PPARGC1B* expression and survival time of patients (Figure 4).

In conclusion, cluster analysis of the 24 genes encoding histone deacetylases and select metabolic regulators in patients with brain glioma tumors allowed us to group genes according to their expression profiles, and facilitated the discrimination of subtypes of primary brain tumors within the same histopathological entity. Furthermore, we found that pairwise Pearson gene-to-gene correlation identified clusters of genes that might cooperate to regulate a yet to be identified signaling pathway(s) in gliomas. We also identified that *HDAC1* and *HDAC3* expression levels were negatively correlated with survival time among glioma patients, whereas the expression of *HDAC4*, *HDAC5*, *HDAC6*, *HDAC11* and *SIRT1* was positively correlated with survival time of patients with gliomas. In addition, HRMAS data revealed that patients with the expression profile *HDAC1*^high^/*HDAC4*^low^/*SIRT1*^low^ present a decreased GPChol/PChol ratio, a metabolomic hallmark of aggressive brain tumors, while those with a *HDAC1*^NC^/*HDAC4*^high^/*SIRT1*^high^ expression profile had significantly higher GPChol/PChol ratio associated with low grade tumors. Concomitant inhibition of *HDAC1* and activation of *HDAC4/SIRT1* may be salutary in restraining highly aggressive tumor progression and may improve survival time in glioma patients.

A better understanding of how HDACs and metabolic cofactors interact to regulate important genes involved in the pathogenesis of high grade gliomas will help design new molecular therapeutic agents to improve the survival of patients and perhaps even cure these deadly diseases. Another challenge is to unravel why patients belonging to the same histo-pathological entity express differentially a subset of HDACs and cofactors, and how this difference in gene expression influence the aggressiveness of the tumor and thereby survival of patients. Our study has begun to answer these questions, thereby paving the way to the establishment of a molecular « fingerprint » of gliomas as a guide to new therapeutic approaches.

## Patients and Methods

### Patients

Patients undergoing surgical resection of brain tumors were from the Department of Neurosurgery (Strasbourg University Hospital). Informed written consent was obtained from all patients studied and the study complies with the Helsinki declaration. The protocol was approved by the ethical committee of protection of persons (“*Comité de Protection des Personnes”*) at the Strasbourg University Hospital (CPP approval number 03/100). Cerebral glioma tumor samples from 50 patients, classified on the basis of the four (I–IV) histo-prognostic grades established by the World Health Organization (WHO), were compared with 15 non-tumoral brain samples from patients operated for epilepsia (CO; n = 15, 30.8 ± 3.2 yrs). Tumor types included grade II oligodendrogliomas (ODII; n = 9, 39.1 ± 3.5 yrs), grade III oligodendrogliomas III (ODIII; n = 22, 44.6 ± 2.5 yrs) and glioblastomas (GL/grade IV; n = 19, 57.6 ± 2.9 yrs). The survival time of operated patients was followed from August 2003 until November 2013, during which 40 patients died (ODII: 5, ODIII: 20, GL: 15).

### qRT-PCR analysis for gene expression profile and cluster analysis

HDAC and NR-cofactor gene expression profiles were established by qRT-PCR on a Lightcycler LC480 (Roche®) and then hierarchically clustered as described^46^. The primer sets used for qRT-PCR analysis are shown in Table S1.

### ^1^H-high resonance magic angle spectroscopy ( ^1^H-HRMAS) analysis

Brain tumor samples were collected with minimum ischemic delays after resection (average time 5 min) and snap-frozen in liquid nitrogen before being stored at −80°C. Biopsy samples (About 20 mg) were prepared at −10°C and introduced into a disposable 30 μl KelF insert. HRMAS spectra were recorded on a Bruker Avance III 500 spectrometer operating at a proton frequency of 500.13 MHz and equipped with a 4 mm double resonance ( ^1^H, ^13^C) gradient HRMAS probe as described^47^.

### Statistics

For gene expression levels, the differences between tumors and control samples were considered statistically significant at p < 0.05 using unpaired t-test. The gene expression level was correlated with survival time of patients using regression analysis. Regarding the survival time, the GL/ODII and ODIII/ODII comparisons were considered statistically significant at p < 0.05 using Mann-Whitney test and the GL/ODIII comparison using an unpaired t-test. Cluster analysis and Pearson's correlation coefficients were determined using TMEV 4.1.01 software (www.tm4.org). Boxplots were generated using the R software and GraphPad Prism 6.0. Multiple comparisons of metabolite quantification among ODII, ODIII and GL in HRMAS analysis were considered statistically significant at p < 0.05 using ANOVA followed by Kruskal-Wallis test.

## Author Contributions

N.D.Y. and J.A. designed experiments. N.D.Y., S.F. and F.M.M. performed experiments. S.C., G.N. and I.J.N. contributed tools and reagents. N.D.Y., S.F., F.M.M., S.H. and J.A. analyzed data. N.D.Y., S.H. and J.A. wrote the manuscript.

## Supplementary Material

Supplementary Information supplementary information
