# Supplementary material for: Gene Expression Mapping of Histone Deacetylases and Co-factors, and Correlation with Survival Time and 1H-HRMAS Metabolomic Profile in Human Gliomas
Source: Sci Rep. 2015 Mar 20;5:9087. doi: 10.1038/srep09087 (PMC4367156; doi:10.1038/srep09087)
Supplement: Supplementary Information [file srep09087-s1.pdf]

Supplemental information

Manuscript : **Gene Expression Mapping of Histone Deacetylases and Co-factors, and Correlation with Survival Time and 1H-HRMAS Metabolomic Profile in Human Gliomas**

By Nassim DALI-YOUCEF, Sébastien FROELICH, François-Marie MOUSSALLIEH, Salvatore CHIBBARO, Georges NOËL, Izzie J. NAMER, Sami HEIKKINEN & Johan AUWERX.

Table S1 : Human primers used for quantitative real-time PCR.

	Forward 5' ➔ 3'	Reverse 5' ➔ 3'
18S	-GGG AGC CTG AGA AAC GGC-	-GGT CGG GAG TGG GTA ATT TG-
HDAC1	-GGA AAT CTA TCG CCC TCA CA-	-CTC GGA CTT CTT TGC ATG GT-
HDAC2	-GAC AGG GTC ATC CCA TGA AG-	-GTC TTA TTG ACC GTA GAA ATT TGA T-
HDAC3	-ACG TGG GCA ACT TCC ACT AC-	-GAC TCT TGG TGA AGC CTT GC-
HDAC4	-AGC GTG AGC AAG ATC CTC AT-	-GCC AAG TAC TCA GCG TCT CC-
HDAC5	-GTG ACA CCG TGT GGA ATG AG-	-AGT CCA CGA TGA GGA CCT TG-
HDAC6	-AAG TAG GCA GAA CCC CCA GT-	-GTG CTT CAG CCT CAA GGT TC-
HDAC7	-CCC AGC AAA CCT TCT ACC AA-	-AAG CAG CCA GGT ACT CAG GA-
HDAC8	-GGT GAC GTG TCT GAT GTT GG-	-AGC TCC CAG CTG TAA GAC CA-
HDAC9	-CCT TCG AAA AAC TGC CTC TG-	-GAC CAG AGC CTG GAG AAC TG-
HDAC10	-TCC ACC CGA GTA CCT TTC AC-	-GAT CCT GTG TAG CCC GTG TT-
HDAC11	-TCT TCC TCC CCA ACT TCC CTT-	-CTC CAC ACG CTC AAA CAG AA-
SIRT1	-GCA GAT TAG TAG GCG GCT TG-	-TCT GGC ATG TCC CAC TAT CA-
SIRT2	-GAG GCC AGG ACA ACA GAG AG-	-TAG AGA TTT GCT GGG GTT GG-
SIRT3	-CAT GAG CTG CAG TGA CTG GT-	-GAG CTT GCC GTT CAA CTA GG-
SIRT4	-CAG CAA GTC CTC CTC TGG AC-	-CCA GCC TAC GAA GTT TCT CG-
SIRT5	-TTC AAA GGC AGA AAC CAA CC-	-CTA ATG CAA AAG CAG CCA CA-
SIRT6	-CCA AGT TCG ACA CCA CCT TT-	-CGG ACG TAC TGC GTC TTA CA-
SIRT7	-CGC CAA ATA CTT GGT CGT CT-	-GTG ATG CTC ATG TGG GTG AG-
PPARGC1A	-CCT GCA TGA GTG TGT GCT CT-	-GCA AAG AGG CTG GTC TTC AC-

PPARGC1B	-AGT GGT GCC CAG AGA ACT CA-	-CGA AGC TGA GGT GCA TGA TA-
NCOR1	-CAT TGC TAG GAG TGA GCA TGA-	-TAG GTG GAA TCA CAG AGA GCT-
NCOR2	-TCA TCT ACG ACG AGA ACC GG-	-ATT GTT TCC GAG CGT GAT TC-
NRIP1	-GTG GAA CAA AGG TCA TGA GTG A-	-CTC GAG AAT ACT GCT GCA AAT G-
RB1	-CAG CAA GTG ATC AAC CTT CA-	-ATT TCG ACA CAA CCC TGT CC-

Table S2 : P-values of 18S normalized expression levels of HDACs and NR co-factors in different brain tumors versus control samples. $p < 0.01$, bold red; $0.01 \le p < 0.05$, red; ≥ 0.05, black.

Class I HDACs

Tumor type	*HDAC1*	*HDAC2*	*HDAC3*	*HDAC8*
GL	**9.88E-06**	5.49E-01	**9.27E-06**	3.61E-01
ODII	9.29E-02	4.17E-01	1.97E-01	6.45E-01
ODIII	**4.43E-05**	5.06E-02	3.28E-02	7.61E-01

Class IV HDACs

Tumor type	*HDAC11*
GL	**7.96E-06**
ODII	7.05E-02
ODIII	**3.03E-03**

Class II HDACs

Tumor type	*HDAC4*	*HDAC5*	*HDAC6*	*HDAC7*	*HDAC9*	*HDAC10*
GL	9.83E-01	**7.97E-08**	**2.24E-04**	**5.00E-07**	4.71E-01	**1.12E-04**
ODII	2.09E-02	1.60E-01	1.64E-02	9.30E-02	7.93E-01	2.28E-01
ODIII	1.46E-02	**5.06E-03**	**1.05E-05**	**1.60E-04**	4.08E-01	**7.34E-06**

Class III HDACs

Tumor type	*SIRT1*	*SIRT2*	*SIRT3*	*SIRT4*	*SIRT5*	*SIRT6*	*SIRT7*
GL	2.51E-01	9.11E-02	1.62E-02	6.65E-02	1.88E-01	1.63E-02	9.50E-02
ODII	2.92E-02	3.99E-01	7.97E-01	1.00E-01	7.14E-01	**5.21E-03**	8.25E-01
ODIII	**8.55E-03**	5.12E-01	2.70E-01	3.15E-01	8.15E-01	**7.98E-04**	1.62E-01

Transcriptional co-factors

Tumor type	*PPARGC1A*	*PPARGC1B*	*NCOR1*	*NCOR2*	*NRIP1*	*RB1*
GL	1.95E-02	**8.01E-08**	4.09E-01	9.11E-02	1.34E-01	**2.38E-03**
ODII	3.84E-01	3.21E-02	9.50E-01	1.05E-01	4.77E-01	6.78E-02
ODIII	1.93E-02	**4.58E-06**	1.93E-01	**7.08E-05**	1.67E-01	**1.97E-03**
